# Targeting metabolic dysfunction-associated steatotic liver disease with phytosomal silymarin and piperine: A natural alternative to fenofibrate in a rat model

**DOI:** 10.1007/s00210-025-04752-1

**Published:** 2025-11-06

**Authors:** Magdy Fouad Tawfik, Yasmin Sayed Hussein, Amany Helmy Hasanin, Walaa Baher, Nashwa El-Khazragy, Farouk Guindi Moawad, Mawada Abou El-Khair, Salwa M. El-Sayed

**Affiliations:** 1 Department of Agricultural Biochemistry, Faculty of Agriculture, Ain Shams University, Cairo, Egypt; 2 Department of Pharmacology, Faculty of Medicine, Ain Shams University, Cairo, Egypt; 3 Department of Histology & Cell Biology, Faculty of Medicine, Ain Shams University, Cairo, Egypt; 4 Department of Clinical Pathology-Hematology and AinShams Medical Research Institute (MASRI), Faculty of Medicine, Ain Shams University, Cairo, 11566 Egypt; 5 Department of Biotechnology, Faculty of Agriculture, Cairo University, Cairo, Egypt

**Keywords:** MASLD, MASH, Steatotic liver disease, Metabolic dysfunction, Silymarin, Piperine, Lecithin, Phytosome

## Abstract

Metabolic dysfunction-associated steatotic liver disease (MASLD) is an increasing global health concern with limited effective therapies. Silymarin exhibits hepatoprotective properties, but its utility is limited by poor bioavailability. This study evaluated a novel phytosomal silymarin formulation co-administered with piperine and lecithin, compared to fenofibrate, in an oxytetracycline-induced MASLD rat model. Twenty-five male Wistar rats were allocated into five groups: healthy control, MASLD control, fenofibrate (100 mg/kg), silymarin (500 mg/kg), or silymarin–piperine–lecithin phytocomplex (500/100/3 mg/kg) for 90 days. Parameters evaluated included serum liver enzymes, lipid profiles, hepatic histology, and hepatic PPAR-α expression. The phytocomplex formulation significantly reduced serum ALT, AST, ALP, and GGT, improved lipid profiles, and restored hepatic architecture, with MASLD-AS and PPAR-α expression demonstrating a marked reduction in hepatic injury and oxidative stress. The phytocomplex outperformed both fenofibrate and silymarin alone, likely due to enhanced bioavailability and synergistic antioxidant action comparable to fenofibrate treatment. This study demonstrates the potential of a phytosomal silymarin–piperine–lecithin complex as a natural therapeutic avenue for MASLD, outperforming a conventional lipid-lowering agent in this animal model. Future clinical and pharmacokinetic studies are warranted.

## Introduction

Metabolic dysfunction-associated steatotic liver disease (MASLD), as recently codified by international consensus, comprises the most prevalent chronic liver disorder (Teng et al. 2023; Wong et al. 2023), closely linked to the global rise in obesity, insulin resistance (Seydel et al. 2016), metabolic syndrome (Radu et al. 2023), and type 2 diabetes mellitus (Pouwels et al. 2022; Oladipupo et al. 2025). MASLD encompasses hepatic steatosis due to metabolic dysfunction and can progress to metabolic dysfunction-associated steatohepatitis (MASH), (Pierantonelli and Svegliati-Baroni 2019) an aggressive form characterized by hepatocellular injury, inflammation, and varying degrees of fibrosis (Younossi 2019). MASH can progress to cirrhosis(Pierantonelli and Svegliati-Baroni 2019), liver failure, and hepatocellular carcinoma (HCC), significantly increasing liver-related morbidity and mortality (Samy et al. 2024). Globally, it is estimated that MASLD affects approximately 25–30% of the adult population, with MASH present in a significant subset (Ye et al. 2020; Younossi and Henry 2021; Younossi et al. 2023). Increased mortality in affected patients is due to both hepatic and cardiovascular complications (Duell et al. 2022; Dong et al. 2024), underscoring the urgent need for therapeutic advances (Mantovani et al. 2020).

**Fig. 1:**
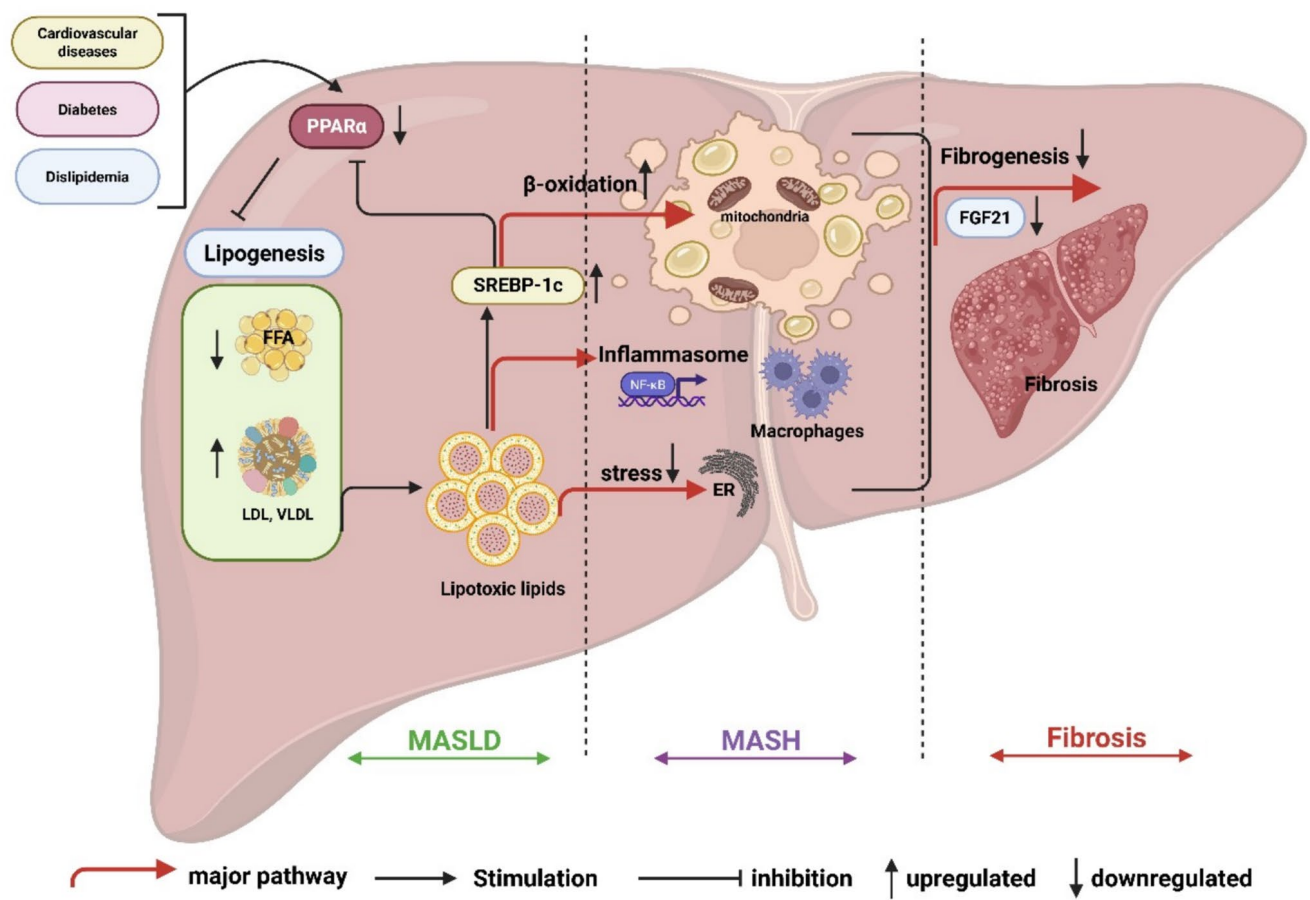
Pathogenic progression of MASLD to MASH and liver fibrosis: Central role of PPAR-α downregulation and protective modulation by FGF21. A schematic diagram depicts the molecular cascade underlying the progression of MASLD to MASH and liver fibrosis, with a focus on *PPAR-α* downregulation and the counter-regulatory role of *FGF21*. Loss of *PPAR-α* activity results in impaired fatty acid β-oxidation, leading to hepatic steatosis, lipotoxicity, and oxidative stress due to reduced expression of key antioxidant enzymes. These disturbances promote hepatocellular injury, ER stress, and activation of pro-inflammatory signaling pathways, facilitating the transition from simple steatosis to MASH. Persistent inflammation and injury activate Kupffer cells through profibrotic mediators, particularly TGF-β1, resulting in fibrogenesis. Importantly, *FGF21*, a downstream target of *PPAR-α*, serves as a protective endocrine regulator that mitigates fibrosis. *FGF21* exerts anti-inflammatory, antioxidative, and antifibrotic effects by suppressing *TGF-β1*/*Smad* signaling, inhibiting HSC activation, and restoring metabolic homeostasis. In states of *PPAR-α* deficiency, reduced *FGF21* expression contributes to unchecked fibrogenic progression. **Abbreviations:** PPAR-α: Peroxisome proliferator-activated receptor alpha, FGF21: Fibroblast Growth Factor 21, HSC: *hepatic stellate cells*, *TGF-β1***:** Transforming growth factor**-***β1,* MASLD: Metabolic dysfunction-associated steatotic liver disease, MASH**:** metabolic dysfunction-associated steatohepatitis

Among molecular pathways, Peroxisome proliferator-activated receptor alpha (PPARα) regulates lipid metabolism and inflammation in the liver (Lin et al. 2022), and its downregulation is implicated in disease progression (Silva and Peixoto 2018). Fibroblast growth factor 21 (FGF21), downstream of PPAR-α, is an important protective hormone against fibrosis (Rusli et al. 2016). Figure 1 Illustrates the mechanistic pathway of progression of MASLD to MASH and liver fibrosis.

Current pharmacological approaches are limited: until recently, no FDA- or EMA-approved drugs specifically target MASLD/MASH (Mantovani et al. 2020; Tidwell et al. 2023). In March 2024, the FDA approved Resmetirom (a thyroid hormone receptor-β agonist) as the first pharmacological treatment for MASH (Keam 2024). Lifestyle change is currently the mainstay, but has variable efficacy and adherence. (Genua and Cusi 2024; Savari and Mard 2024). This has renewed interest in natural compounds. *Silymarin*, an extract of *Silybum marianum (milk thistle)*, displays hepatoprotective, antioxidant, anti-inflammatory, and antifibrotic effects, but its impact is hampered by poor oral bioavailability(Mengesha et al. 2021; Pandey et al. 2023).

*Silymarin* is a standardized extract composed of several flavonolignans, primarily silybin (also known as *silibinin*), *silydianin*, and *silychristin*, which together account for most of its pharmacological activity (Koushki et al. 2023; Jaffar et al. 2024a, b). Its hepatoprotective properties have been attributed to multiple mechanisms, including antioxidant activity, free radical scavenging (Dhande et al. 2024), membrane-stabilizing effects, anti-inflammatory action (Surai et al. 2024; Vajdi et al. 2025), and modulation of hepatic stellate cells and fibrogenesis (Taleb et al. 2018; Wadhwa et al. 2022). Silymarin has also been reported to regulate lipid metabolism, reduce hepatic fat accumulation, and improve insulin sensitivity (De Freitas et al. 2024; Guo et al. 2024). Despite its therapeutic potential, the clinical utility of silymarin is limited by its poor oral bioavailability, mainly due to low solubility and extensive first-pass metabolism (Sornsuvit et al. 2018; Song et al. 2021).

Formulation strategies such as phytosome technology have been explored to overcome these limitations (Lee et al. 2024). Phytosomes are complexes of natural active compounds and phospholipids, most notably lecithin, a naturally occurring phospholipid primarily composed of phosphatidylcholine (De Freitas et al. 2024; Lee et al. 2024). The integration of silymarin with lecithin enhances its lipophilicity, allowing for improved solubility in biological membranes and increased gastrointestinal absorption (Talebi et al. 2025). This improved pharmacokinetic profile translates into enhanced therapeutic efficacy, making silymarin–lecithin complexes (phytosomes) a promising delivery system for liver-targeted therapy (Lu et al. 2019; Gohari Mahmoudabad et al. 2023).

Moreover, the incorporation of *piperine*, a bioenhancer derived from *Piper nigrum* (black pepper), further augments the bioavailability of *silymarin* (Chaudhri and Jain 2023). *Piperine*, an alkaloid responsible for the pungency of black pepper, has been shown to inhibit hepatic and intestinal glucuronidation, enhance gastrointestinal absorption, and increase the plasma concentration of co-administered drugs and phytochemicals (Tiwari et al. 2020). Its role as a bioenhancer in polyherbal formulations has been extensively documented, particularly in improving the systemic availability of poorly absorbable compounds such as curcumin and silymarin (Tripathi et al. 2022). *Piperine* also exhibits anti-inflammatory, antioxidant, and lipid-lowering properties, which may provide synergistic benefits in the context of MASLD (Pratti et al. 2024).

On the other hand, *fenofibrate*, a well-established lipid-lowering agent belonging to the fibrate class, is frequently utilized in the management of hyperlipidemia and hypertriglyceridemia (Nguyen and Park 2022; Deerochanawong et al. 2024). Chemically, fenofibrate is an isopropyl ester of fenofibric acid, which acts as a ligand for peroxisome proliferator-activated receptor-alpha (PPAR-α). Activation of PPAR-α promotes fatty acid oxidation, reduces hepatic triglyceride synthesis, and improves plasma lipid profiles (Lee et al. 2013). In preclinical and clinical studies, fenofibrate has demonstrated beneficial effects in reducing hepatic steatosis and inflammation, making it a candidate for MASLD management (Vecera et al. 2022; Zhang et al. 2023). However, concerns regarding long-term safety, renal effects, and modest efficacy have limited its widespread adoption in MASH therapy (Jiang et al. 2022a, b; Mahmoudi et al. 2022a, b).

**Fig. 2:**
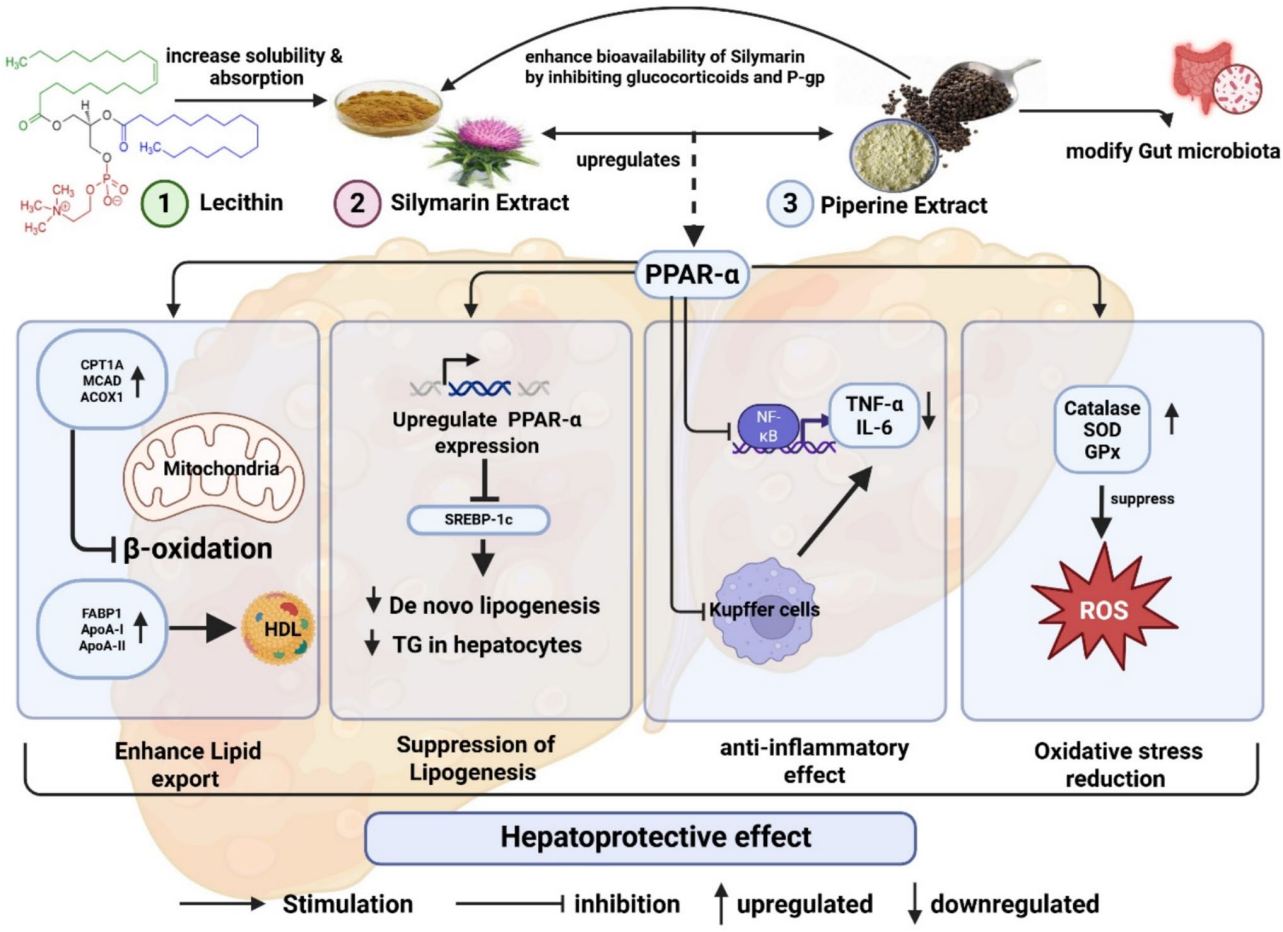
Hepatoprotective mechanisms of the silymarin–lecithin–piperine phytocomplex in Metabolic dysfunction-associated steatotic Liver Disease (MASLD). A schematic diagram illustrates the proposed molecular pathways through which the combined administration of *silymarin*, *lecithin*, and *piperine* exerts hepatoprotective effects in MASLD. In hepatocytes, both silymarin and piperine enhance the expression of *PPAR-α*, a central regulator of lipid metabolism and β-oxidation. This activation occurs via four interconnected mechanisms: (1) promotion of lipid export and fatty acid oxidation through upregulation of *CPT1A*, ACOX1, MCAD, and increased HDL synthesis via elevated expression of *FABP1, ApoA-I*, and *ApoA-II*; (2) suppression of de novo lipogenesis by downregulating key transcription factors *SREBP-1c* and *ChREBP*; (3) attenuation of hepatic inflammation through inhibition of *NF-κB* signaling and suppression of Kupffer cell activity, leading to decreased production of pro-inflammatory cytokines *IL-6* and *TNF-α*; and (4) reduction of oxidative stress by upregulating the expression of antioxidant enzymes including catalase, SOD, and GPx, while concurrently decreasing reactive oxygen species (ROS) generation. In addition, piperine modulates the gut–liver axis by correcting intestinal dysbiosis, thereby reducing endotoxin-mediated hepatic inflammation. Lecithin enhances the lipophilicity and membrane permeability of silymarin, improving its solubility, gastrointestinal absorption, and systemic bioavailability. The integration of silymarin–lecithin complexes with piperine thus represents a promising strategy to improve the pharmacokinetic profile and therapeutic efficacy of phytotherapeutic interventions in MASLD. Abbreviations: PPAR-α: peroxisome proliferator-activated receptor alpha, CPT1A: carnitine palmitoyl transferase 1 A, ACOX1: acyl-CoA oxidase 1, MCAD: medium-chain acyl-CoA dehydrogenase, FABP1: fatty acid-binding protein 1, ApoA-I: apolipoprotein A-I, SREBP-1c: sterol regulatory element-binding protein-1c, ChREBP: carbohydrate-responsive element-binding protein, HDL: high-density lipoprotein, NF-κB: nuclear factor kappa B, IL6: interleukin-6, TNF-α: tumor necrosis factor-alpha, SOD: superoxide dismutase, GPx: glutathione peroxidase, ROS: reactive oxygen species

While *Silymarin* is known for its antioxidant and anti-inflammatory properties, its poor oral bioavailability has limited its therapeutic potential (Jaffar et al. 2024a, b). Recent advances in phytosome technology and bioenhancer co-administration may overcome this barrier, yet there remains a critical gap in knowledge regarding how such enhanced natural formulations compare directly to conventional pharmacological agents like fenofibrate (Vecera et al. 2022) (Mahmoudi et al. 2022a, b). Figure 2 illustrates the hepatoprotective mechanisms of the Silymarin–lecithin–piperine phytocomplex in MASLD.

To date, few studies have directly compared optimized silymarin phytocomplexes with piperine and lecithin with conventional therapies for MASLD. The addition of piperine, which acts as a bioenhancer by reducing metabolic clearance, and lecithin serves as a phospholipid carrier that improves the solubility and membrane permeability of silymarin. Incorporating lecithin into the formulation facilitates phytosome formation, thereby enhancing intestinal absorption and systemic bioavailability of silymarin. This study addresses this gap using a rat model.

## Materials and methods

### Preparation of silymarin extract

#### Preparation and standardization of silymarin extract

A total of 500 g of dried milk thistle (*Silybum marianum*) seeds were obtained from Agricultural Seeds, Spices and Medicinal Plants Co. (Al-Azhar Street, Cairo, Egypt). The seeds were finely ground using a laboratory grinder and subjected to two successive rounds of cold maceration in 70% ethanol using a 2:1 solvent-to-solid ratio (v/w). Each extraction was performed over 24 h at room temperature (25 ± 2 °C) with intermittent stirring. Following each extraction cycle, the suspensions were filtered using Whatman No. 1 filter paper to remove solid residues. The combined filtrates were then concentrated under reduced pressure using a vacuum rotary evaporator (***Heidolph, Germany***) at 40 °C until most of the ethanol was evaporated. The remaining semi-solid extract was dried to yield the crude silymarin extract. The dried extract was carefully weighed and stored in amber-glass containers at − 20 °C until further use (Shafiei et al. 2024).

#### Phytochemical screening

The crude extract was screened for the presence of major bioactive constituents. Tests were conducted for flavonoids (Shinoda test), phenolic compounds (Ferric chloride test), tannins, alkaloids (Dragendorff's and Mayer's tests), saponins (froth test), and terpenoids. The extract tested strongly positive for flavonolignans and phenolic compounds, confirming the expected silymarin profile (Muchiri and van Breemen 2024).

#### In vivo* experimental design*

A total of 25 male albino rats of the Wistar strain, each weighing approximately 100 ± 10 g, were obtained from the ***Animal House Unit at the National Research Center, Dokki, Giza***, ***Egypt***. Only male rats were included in order to minimize variability related to estrogen’s hormonal effects, which are known to influence hepatic lipid metabolism. The animals were housed under controlled environmental conditions (temperature 23 ± 2 °C, relative humidity 50% ± 10%) with a 12-h light/dark cycle. Prior to the experiment, all rats were acclimatized for one week and fed a standard basal diet composed of 70% corn starch, 10% casein, 10% corn oil, 4% salt mixture, 1% vitamin mixture, and 5% cellulose. Animals had ad libitum access to food and water throughout the study duration (Zhou et al. 2023).

The rats were randomly assigned into five groups (n = 5 per group), with non-overlapping treatment protocols over a 90-day period as follows:

- ***Group 1 (Normal Control):*** Fed only the basal diet, with no drug interventions.
- ***Group 2 (MASLD Model – Negative Control):*** Received intraperitoneal injections of oxytetracycline (120 mg/kg body weight/day; Sigma-Aldrich, St. Louis, MO, USA) for three consecutive days to induce Metabolic Dysfunction-Associated Steatotic Liver Disease (MASLD), followed by a basal diet without treatment.
- ***Group 3 (Fenofibrate – Positive Control):*** Received intraperitoneal injections of oxytetracycline (120 mg/kg/day for 3 days) to induce MASLD, followed by oral administration of fenofibrate (100 mg/kg/day; Sigma-Aldrich) via oral gavage alongside the basal diet for 90 days
- ***Group 4 (Silymarin Extract Only):*** Received intraperitoneal injections of oxytetracycline (120 mg/kg/day for 3 days) to induce MASLD, followed by treatment with 500 mg/kg/day of crude milk thistle (*Silybum marianum*) extract (prepared as described above), administered every other day orally by gastric tube, along with basal diet for 90 days.
- ***Group 5 (Enhanced Phytocomplex):*** Received intraperitoneal injections of oxytetracycline (120 mg/kg/day for 3 days) to induce MASLD, followed by combined oral administration of silymarin extract (500 mg/kg), lecithin (100 mg/kg; Alfa Aesar, Germany), and piperine (3 mg/kg; Sigma-Aldrich), administered every other day for 90 days by gastric tube along with the basal diet for 90 days.
- In addition, three supplementary groups were included to assess the contribution of individual components: silymarin + piperine, silymarin + lecithin, and the full silymarin–piperine–lecithin phytocomplex.

All dosing volumes were adjusted according to individual body weights. The experimental protocol was approved by the ***Ethics Committee of the Faculty of Agriculture, Ain Shams University***, and all procedures conformed to internationally accepted guidelines for the care and use of laboratory animals.

### Sample collection and biochemical analysis

At the end of the 90-day treatment period, all animals were fasted overnight and then anesthetized using intraperitoneal injection of ketamine (80 mg/kg) and xylazine (10 mg/kg). Blood samples were collected via cardiac puncture using sterile syringes and transferred into plain centrifuge tubes. Samples were allowed to clot at room temperature and then centrifuged at 3000 rpm for 15 min to obtain clear serum. The serum was aliquoted and stored at − 20 °C for subsequent biochemical analyses.

Liver function parameters, including serum alanine transaminase (ALT), aspartate transaminase (AST), alkaline phosphatase (ALP), total bilirubin, and albumin levels, were measured using standard enzymatic colorimetric kits (***BioSystems S.A., Barcelona, Spain***) according to the manufacturer's protocols. In addition, lipid profile markers such as total cholesterol, triglycerides, high-density lipoprotein (HDL), and low-density lipoprotein (LDL) were also assessed to evaluate systemic metabolic status (***BioSystems S.A., Barcelona, Spain***)(Wang et al. 2016).

Total hepatic lipid content was quantified from frozen liver tissue using the classical Folch extraction method. Briefly, approximately 200 mg of liver was homogenized in chloroform–methanol (2:1, v/v) at a ratio of 20 volumes of solvent per gram of tissue. The homogenate was filtered, and a 0.9% NaCl solution was added to induce phase separation. After centrifugation, the lower organic phase containing the lipids was carefully collected, dried under a stream of nitrogen, and weighed gravimetrically to determine total lipid mass. Results were expressed as grams of lipid per 100 g of wet liver tissue (Breil et al. 2017).

### Liver tissue collection and histological examination

Immediately following blood collection, the animals were euthanized by cervical dislocation under deep anesthesia. The livers were quickly excised, rinsed in ice-cold normal saline to remove blood residues, and blotted dry. Each liver was weighed, and the liver-to-body weight ratio was calculated. A portion of the liver was fixed in 10% neutral buffered formalin for 48 h, then processed for histopathological examination. Formalin-fixed tissues were embedded in paraffin, sectioned at 5 μm thickness using a rotary microtome, and stained with hematoxylin and eosin (H&E). Additional sections were stained with Masson's trichrome to assess collagen deposition and hepatic fibrosis.

The histological scoring system for MASLD and MASH is based on an integrated assessment of specific liver biopsy features: steatosis, lobular inflammation, hepatocellular ballooning, and fibrosis. Steatosis is graded by the proportion of hepatocytes containing fat droplets, scored as follows: 0: < 5%, 1:5–33%, 2:34–66%, 3: > 66%. Lobular inflammation reflects the number of inflammatory foci per microscopic field (0: none; 1: < 2 foci/20x; 2: 2–4 foci/20x; 3: > 4 foci/20x). Hepatocellular ballooning (degeneration) is graded from 0 (none), 1 (few), to 2 (many). The overall activity score, commonly called NAS (MASLD Activity Score, but equally applicable to MASLD/MASH), is the unweighted sum of these three components, yielding a total score ranging from 0 to 8. Fibrosis is staged separately (0: none, 1: perivenular/zone 3; 2: periportal and perivenular; 3: bridging fibrosis; 4: cirrhosis). MASH diagnosis requires the simultaneous presence of steatosis, inflammation, and ballooning, typically corresponding to higher total scores (Tamaki et al. 2021; Chan et al. 2023).

### Immunohistochemical detection of PPAR-α expression

Immunohistochemical (IHC) staining was performed to evaluate the expression of peroxisome proliferator-activated receptor-alpha (PPAR-α) in liver tissue sections from all experimental groups. Formalin-fixed, paraffin-embedded liver samples were sectioned at 4 µm thickness and mounted on positively charged glass slides. Sections were deparaffinized in xylene and rehydrated through a descending ethanol gradient. Antigen retrieval was carried out by heating the slides in 10 mM citrate buffer (pH 6.0) in a microwave oven for 15 min.

Endogenous peroxidase activity was quenched by incubating the sections in 3% hydrogen peroxide for 10 min at room temperature. Non-specific binding was blocked using 5% normal goat serum for 30 min. The tissue sections were then incubated overnight at 4 °C with the primary antibody against PPAR-α (Rabbit polyclonal anti-PPAR-α, Thermo Fisher Scientific, Cat. No. MA5-37,652), diluted 1:200 in antibody diluent. Following primary antibody incubation, the sections were washed with phosphate-buffered saline (PBS) and incubated with a biotinylated secondary antibody (goat anti-mouse IgG) for 30 min at room temperature.

Signal detection was performed using a streptavidin–horseradish peroxidase (HRP) conjugate and developed with 3,3′-diaminobenzidine (DAB) as the chromogen. Slides were counterstained with Mayer's hematoxylin, dehydrated, cleared, and mounted with coverslips. Negative control slides were processed in parallel by omitting the primary antibody (Mebratie and Dagnaw 2024).

PPAR-α immunoreactivity was assessed under a light microscope. The extent and intensity of nuclear staining were semi-quantitatively scored by a blinded histopathologist using a modified histo-score (H-score) method based on both the percentage of positively stained nuclei and staining intensity (graded 0–3). The final H-score was calculated as:

- H-score = [(% of cells with weak staining × 1) + (% with moderate × 2) + (% with strong × 3)], with a total score range of 0–300 (Mebratie and Dagnaw 2024).

### Statistical analysis

All data were expressed as mean ± standard deviation (SD). Statistical analysis was performed using GraphPad Prism software version 9.0 (GraphPad Software Inc., San Diego, CA, USA). Normality of data distribution was assessed using the Shapiro–Wilk test. For multiple group comparisons, one-way analysis of variance (ANOVA) was applied, followed by Tukey's post hoc test to determine intergroup differences. A value of *p* < 0.05 was considered statistically significant.

## Results

### Effect of treatments on liver function, lipid profile, and hepatic lipid accumulation in oxytetracycline-induced MASLD rats

Table 1 presents the biochemical parameters related to liver function, lipid metabolism, and hepatic fat accumulation across five experimental groups: Healthy control, MASLD control, MASLD + Fenofibrate, MASLD + Milk Thistle Extract, and MASLD + Milk Thistle Extract combined with Piperine and Lecithin.

**Table 1:** Effect of fenofibrate, milk thistle extract, and milk thistle enhanced with piperine and lecithin on liver function enzymes, bilirubin levels, protein profile, serum lipids, and hepatic lipid content in oxytetracycline-induced MASLD in Wistar rats

	Healthy	MASLD	MASLD + Fenofibrate	MASLD + Milk Thistle	MASLD + (Milk Thistle + PIP + LEC)	p-value
ALT (U/L)	29.60 ± 2.54	293.40 ± 3.95^**a**^	274.2 ± 18.85^**a/b**^	56.6 ± 0.87^**a/b**^	40.80 ± 3.07^**a/b**^	0.0001
AST (U/L)	39.10 ± 2.72	304.40 ± 6.48^**a**^	287.2^a^ ± 26.43^**a**^	66.40 ± 1.39^**a/b**^	51.80 ± 2.09^**a/b**^	0.0001
GGT (U/L)	21.2 ± 3.00	80.80 ± 4.46^**a**^	29.80 ± 3.54^**a/b**^	46.40^d^ ± 2.36^**a/b**^	26.10 ± 1.39^**a/b**^	0.0001
ALP (U/L)	96.6 ± 6.77	135.2 ± 3.42^**a**^	256.6 ± 28.38^**a/b**^	146.60 ± 2.14^**a**^	104.8 ± 6.72^**a/b**^	0.0001
Bilirubin Total (mg/dl)	0.39 ± 0.02	1.24 ± 0.25^**a**^	2.58 ± 0.40^**a/b**^	0.40 ± 0.01^**b**^	0.36 ± 0.03^**b**^	0.0001
D. Bilirubin (mg/dl)	0.15 ± 0.02	0.38 ± 0.10	1.09 ± 0.39^**b**^	0.19 ± 0.01	0.18 ± 0.01	0.0001
Indirect bilirubin (mg/dl)	0.23 ± 0.01	0.86 ± 0.19^**a**^	1.49 ± 0.10^**a/b**^	0.21 ± 0.02^**a/b**^	0.18 ± 0.03^**a/b**^	0.0001
Total protein (g/dl)	7.10 ± 0.17	4.37 ± 0.21^**a**^	4.62^e^ ± 0.12^**a**^	6.58 ± 0.17^**a/b**^	6.80 ± 0.11^**a/b**^	0.0001
Albumin (g/dl)	3.82 ± 0.12	2.30 ± 0.16^**a**^	2.46 ± 0.18^**a**^	3.30 ± 0.07^**b**^	3.42 ± 0.07^**b**^	0.001
Globulin (g/dl)	3.28 ± 0.22	2.07 ± 0.38^**a**^	2.16 ± 0.16^**a**^	3.28 ± 0.23^**b**^	3.38 ± 0.13^**b**^	0.01
A/G Ratio	1.19 ± 0.11	1.28 ± 0.42^**a**^	1.17 ± 0.17^**a**^	1.02 ± 0.08^**a**^	1.02 ± 0.05^**a**^	0.002
Total Cholesterol (mg/dl)	96.8 ± 3.72	322 ± 23.38^**a**^	165.2 ± 18.0^**a/b**^	127.0 ± 0.91^**a/b**^	108.4 ± 3.75^**b**^	0.0001
Triglyceride (mg/dl)	85.2 ± 1.24	532.4 ± 29.4^**a**^	285.2 ± 20.41^**a/b**^	150.2 ± 8.40^**a/b**^	113.0 ± 4.9^**a/b**^	0.0001
HDL(mg/dl)	43.0 ± 0.94	33.6 ± 1.85^**a**^	56.80 ± 3.88^**b**^	41.4 ± 0.87^**b**^	47.6 ± 1.94^a/**b**^	0.005
LDL(mg/dl)	36.7 ± 2.26	181.9 ± 30.41^**a**^	51.36 ± 14.03^**a/b**^	55.56 ± 3.70^**a/b**^	38.20 ± 4.88^**b**^	0.001
VLDL(mg/dl)	17.0 ± 0.24	106.4 ± 7.60^**a**^	57.04 ± 5.27^**a/b**^	30.04 ± 2.17^**a/b**^	22.6 ± 1.26^**a/b**^	0.0001
R1 (Total Cholesterol/HDL)	2.25 ± 0.11	9.72 ± 1.15^**a**^	2.89 ± 0.18^**a/b**^	3.07 ± 0.08^**a/b**^	2.29 ± 0.16^**a/b**^	0.001
Total Hepatic Lipids (g/100 g)	2.2 ± 0.22	7.26 ± 0.95^a^	5.82 ± 0.51^a/b^	4.25 ± 0.62^a/b^	3.5 ± 0.29^a/b^	0.001
Hepatic Total Triglyceride (mg/g)	12.6 ± 0.235	56.1 ± 1.524^**a**^	25.28 ± 2.21^**a/b**^	22.12 ± 1.15^**a/b**^	13.12 ± 1.45^**b**^	0.0001
Hepatic Total Cholesterol (mg/g)	5.30 ± 0.08	12.9 ± 0.65^**a**^	9.16 ± 0.24^**a/b**^	6.74 ± 0.023^**a/b**^	5.60 ± 0.152^**b**^	0.0001

*ALT* Alanine transaminases; *AST* Aspartate transaminases; *ALP* Alkaline phosphatase; *bilirubin* direct bilirubin; *A/G* Albumin/globulin ratio; *HDL* High density lipoproteins; *LDL* low-density lipoproteins; *VLDL* very -low density lipoproteins; *Healthy* Treated with saline; *MASLD* Metabolic dysfunction-associated steatotic liver disease; *PIP* Piperine; *MASLD group* Oxytetracycline -induced MASLD model, Superscripts denote significant differences: ^a^:signifcant difference compared to healthy control group (p < 0.05), ^**b**^: significant difference compared to MASLD group (p < 0.05). Data are presented in mean ± SD (n = 5/group). Statistical analysis was performed using one-way ANOVA followed by Tukey's multiple comparison test.

The MASLD control group exhibited a dramatic elevation in liver enzymes, indicating significant hepatocellular injury. Serum ALT and AST levels rose markedly (293.4 ± 3.95 U/L and 304.4 ± 6.48 U/L, respectively) compared to those of the healthy control. Fenofibrate treatment led to a partial reduction in both enzymes, whereas milk thistle extract, particularly when combined with piperine and lecithin, significantly normalized ALT (40.8 ± 3.07 U/L) and AST (51.8 ± 2.09 U/L), approaching healthy levels. Similar trends were observed for GGT and ALP, with the combined phyto-complex group (MASLD + Milk Thistle + PIP + LEC) achieving greater improvements than fenofibrate alone.

Total and direct bilirubin levels were significantly elevated in the MASLD group, reflecting impaired hepatic clearance. These levels were substantially reduced in the milk thistle-treated groups, with the combined phytocomplex formulation outperforming all other interventions (Total bilirubin: 0.36 ± 0.03 mg/dL; direct bilirubin: 0.18 ± 0.01 mg/dL).

Protein metabolism was notably disrupted in MASLD rats, as evidenced by reduced total protein, albumin, and globulin levels. These declines were significantly reversed by both forms of milk thistle treatment. In particular, the combination of milk thistle with piperine and lecithin yielded near-normal protein and albumin values (6.80 ± 0.11 g/dL and 3.42 ± 0.07 g/dL, respectively), whereas fenofibrate was less effective in restoring these parameters. Interestingly, the A/G ratio remained largely unchanged across treatment groups, suggesting proportional restoration of both protein fractions.

In terms of lipid profile, the MASLD group displayed classic dyslipidemia with elevated total cholesterol (322 ± 23.38 mg/dL), triglycerides (532.4 ± 29.4 mg/dL), LDL (181.9 ± 30.41 mg/dL), and VLDL, along with a decrease in HDL levels. Fenofibrate and both milk thistle treatments significantly reduced total cholesterol and triglycerides, with the combined phytocomplex group demonstrating the most favorable effect on all parameters, including restoration of HDL (47.6 ± 1.94 mg/dL) and LDL (38.2 ± 4.88 mg/dL) to levels comparable to the healthy group. The atherogenic index (Total Cholesterol/HDL ratio, R1) also normalized in this group (2.29 ± 0.16), underscoring the cardioprotective potential of the formulation.

Total Hepatic lipids, total triglyceride, and cholesterol contents were markedly elevated in the MASLD group, consistent with steatosis. Treatment with fenofibrate and milk thistle extract led to significant reductions in hepatic lipid accumulation. Again, the combined formulation (milk thistle + piperine + lecithin) yielded the greatest improvement, reducing total hepatic lipids, triglycerides, and cholesterol to near-normal levels (p < 0.05) vs. the MASLD group.

The supplementary groups receiving silymarin with either piperine or lecithin did not show significant differences compared with the silymarin-alone group, indicating that the enhanced efficacy observed was specific to the complete phytocomplex combination.

### Histopathological evaluation of H&E-stained liver sections (Fig. 3)

**Fig. 3:**
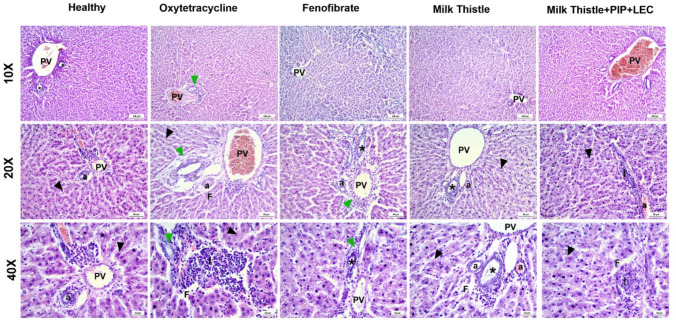
Representative H&E-stained liver sections from each experimental group at three magnifications: 10 ×, 20 ×, and 40 ×. Healthy control: Preserved hepatic architecture with intact portal triad, polyhedral hepatocytes, and clear sinusoidal spaces. Oxytetracycline -MASLD: Severe macrovesicular steatosis, lobular inflammation, hepatocellular ballooning, and fibrosis. MASLD + Fenofibrate: Partial histological improvement with reduced steatosis and inflammation; bridging fibrosis remains. MASLD + Milk Thistle: Marked reduction in fat accumulation and inflammation; hepatocyte morphology and lobular structure improved. MASLD + Milk Thistle + Piperine + Lecithin: Near-complete histological normalization with minimal steatosis, inflammation, or fibrosis, closely resembling a healthy control. PV: portal vein, a: branch of hepatic artery, *: bile duct, black arrowhead: hepatocytes, green arrowhead: periorbital fibrosis, I: inflammatory infiltrate, F: fibrosis. The microscopic examination, stained sections were performed by ***LABOMED Fluorescence microscope LX400***, cat no: 9126000; US and ***LABOMED camera software***, ***USA***. The scale bar and the magnification power are presented on each image

Histological examination of hematoxylin and eosin (H&E)-stained liver sections revealed distinct morphological changes across the experimental groups, reflecting varying degrees of hepatic injury and the therapeutic impact of the applied interventions. In the healthy control group, normal hepatic architecture was observed, with well-organized hepatocyte cords radiating from the portal triad. The portal area showed a clearly defined portal vein (**PV**), a branch of the hepatic artery (**a**), and bile ducts lined by cuboidal epithelial cells (*), all surrounded by minimal fibrous tissue and connective tissue cells (**F**). The surrounding hepatocytes appeared as polyhedral cells with homogeneous acidophilic cytoplasm and vesicular nuclei (**black arrowhead**). Sinusoidal spaces were clearly defined and devoid of inflammatory infiltrates or steatosis (Fig. 3).

In contrast, the MASLD model group (*oxytetracycline*-treated) demonstrated marked histopathological disruptions consistent with steatohepatitis. The portal canal contained a large, congested branch of the portal vein (**PV**) and distorted bile ducts surrounded by fibrous tissue and intense infiltration of inflammatory cells (**green arrowhead**). The hepatic parenchyma showed diffuse macrovesicular steatosis, with hepatocytes distended by large cytoplasmic lipid vacuoles displacing the nuclei to the periphery (black arrowhead). Numerous foci of lobular inflammation (I) were observed, characterized by mononuclear cell infiltration. Additional findings included hepatocellular ballooning, dilated sinusoids, and occasional foci of necrosis. Notably, prominent fibrotic strands (**F**) were observed within the portal and periportal regions, indicative of active metabolic steatohepatitis (MASH) and confirming successful disease induction (Fig. 3).

In the fenofibrate-treated group (Fig. 3), partial histological improvement was noted. The portal triad components, including the portal vein (**PV**), hepatic artery branch (**a**), and bile ducts (*), were still surrounded by considerable fibrous tissue and infiltrating inflammatory cells (**green arrowhead**). Hepatocytes exhibited reduced steatosis, with a noticeable shift from macrovesicular to microvesicular lipid accumulation in several hepatic lobules. Ballooned hepatocytes (**black arrowhead**) were less frequent, and inflammatory foci (I) were diminished but still present. However, fibrous septa infiltrated by inflammatory cells extended between portal tracts, forming bridging fibrosis (**F**). Despite these improvements, complete resolution of steatohepatitis was not achieved, as evidenced by persistent areas of parenchymal disruption.

Liver sections from the group treated with milk thistle extract alone demonstrated notable histological restoration. The portal canal contained a congested branch of the portal vein (**PV**) and bile ducts(*), surrounded by a modest amount of fibrous tissue (**F**) and sparse inflammatory cells (**green arrowhead**). Steatosis was significantly reduced, and overall lobular architecture appeared preserved. Periportal hepatocytes showed only minimal cytoplasmic vacuolization due to small lipid droplets (**black arrowhead**). Mild inflammatory infiltrates (I) were seen in limited foci*.* The hepatocytes displayed centrally located nuclei and intact cytoplasmic integrity, reflecting early regenerative activity. Minimal fibrotic areas (**F**) were observed, suggesting attenuation of disease progression (Fig. 3).

Remarkably, the group treated with milk thistle extract combined with piperine and lecithin exhibited near-complete histological normalization. Hepatic cords were orderly and well aligned, and the portal area, including the portal vein (**PV**), appeared structurally intact. Hepatocytes were polygonal in shape with granular eosinophilic cytoplasm and centrally located vesicular nuclei (**black arrowhead**). Fat vacuoles were rare or absent, and lobular inflammatory infiltrates (I) were either minimal or not observed. Fibrosis (**F**), particularly in the periportal region, was nearly absent. No signs of hepatocellular ballooning or necrosis were detected. These sections closely resembled those of the healthy control group, indicating robust hepatoprotection and histological recovery afforded by the combined Phyto complex treatment (Fig. 3).

### Histological activity scoring reveals significant improvement in hepatic injury following milk thistle and phytocomplex treatment (Table 2 and Fig. 4)

**Table 2:** Metabolic dysfunction-associated steatotic liver disease Activity Score (NAS) in H&E-Stained Liver Sections Across Experimental Groups

Group	Steatosis Score (0–3)	Lobular Inflammation Score (0–3)	Ballooning Score (0–2)	Total NAS (0–8)
Healthy Control	0.2 (0.1)^**b**^	0 (0)^**b**^	0 (0)b	0.2 (0.1)^**b**^
MASLD Control	3 (2.0)^**a**^	2.8 (2.5)^**a**^	2 (1.5)^**a**^	7.8 (7.2)^**a**^
MASLD + Fenofibrate	1.8 (1.5)^**a/b**^	1.4 (1.2)^**a/b**^	1.2 (0.9)^**a/b**^	4.4 (4.2)^**a/b**^
MASLD + Milk Thistle	1.5 (1.0)^**a/b**^	0.8 (0.6)^**a/b**^	0.6 (0.4)^**a/b**^	2.4 (2.0)^**a/b**^
MASLD + Milk Thistle + Piperine + Lecithin	0.4 (0.2)^a/b^	0.2 (0.1)^**a/b**^	0.2 (0.1)^**a/b**^	0.8 (0.6)^**a/b**^

Values are presented as the median value (25% percentile)of the histological scores for steatosis (0–3), lobular inflammation (0–3), hepatocellular ballooning (0–2), and the total NAS (0–8) in each group. Superscripts denote significant differences: ^a^: Significant difference compared to the healthy control group (p < 0.05);^b^: Significant difference compared to the MASLD group (p < 0.05);^a/b^: Significant difference compared to both.

**Fig. 4:**
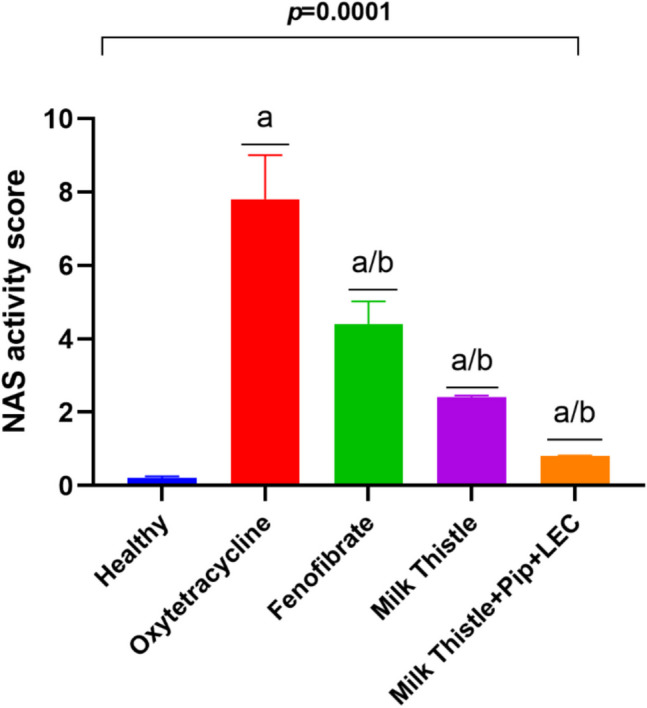
Bar chart illustrating the calculated NAS score "Steatosis, ballooning, lobular inflammation", across groups. Data are expressed as median (range) (n = 5); statistical analysis was performed using one-way ANOVA followed by Tukey's post hoc test. Superscripts denote significant differences: a: significantly different from healthy control (p < 0.01); b: significantly different from Oxytetracycline-induced MASLD (p < 0.01)

**Fig. 5:**
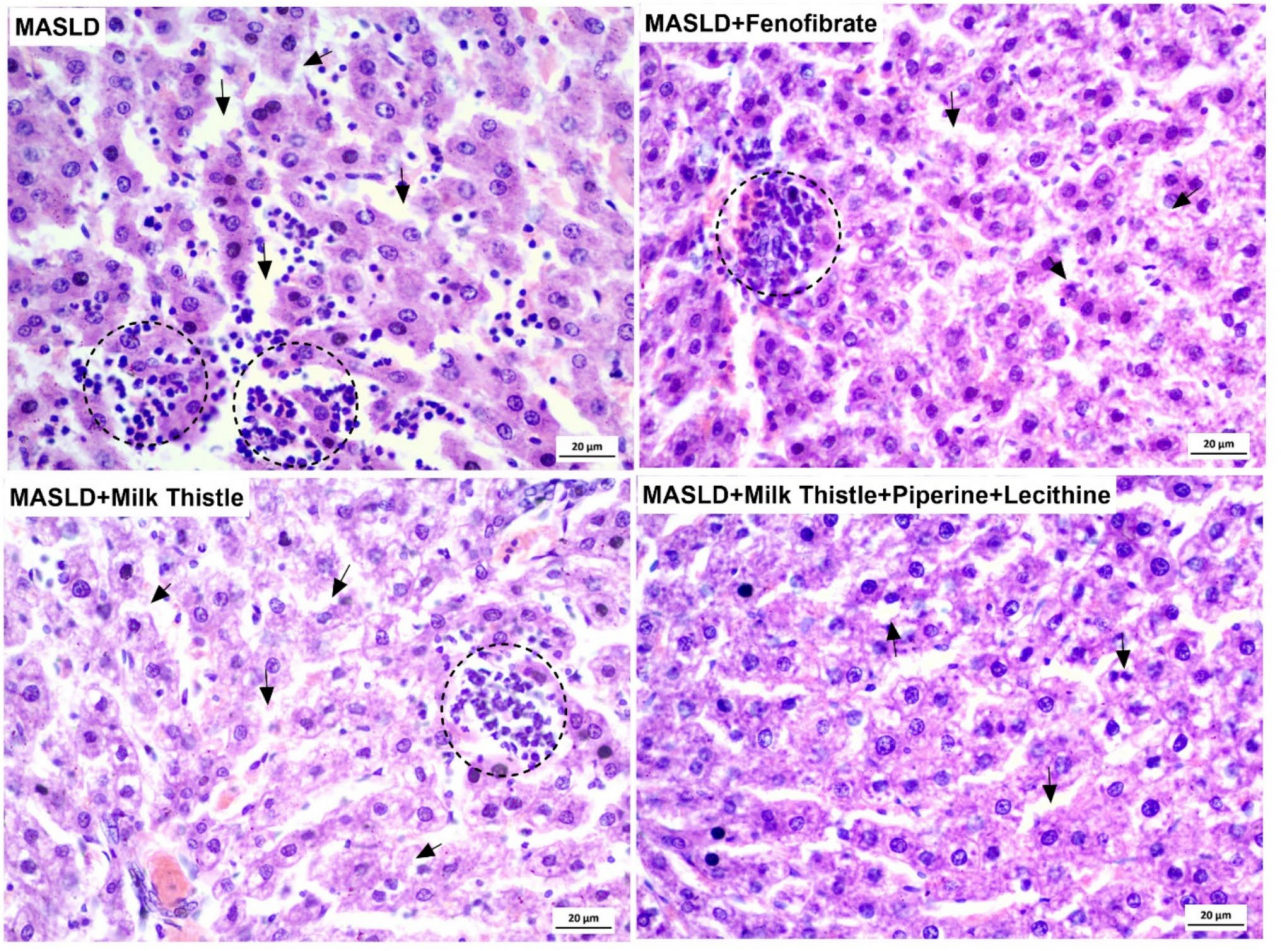
Representative histological images and histological activity scores in liver sections of MASLD-induced rats. Hematoxylin and eosin (H&E) stained liver sections showing macrovesicular and microvesicular steatosis (black arrows) and inflammatory foci (black dotted circles). The MASLD control group shows prominent macrovesicular steatosis and multiple inflammatory foci. The MASLD + fenofibrate group exhibits reduced steatosis and inflammation. The MASLD + milk thistle group demonstrates mild microvesicular steatosis and a few inflammatory foci. MASLD + milk thistle + piperine + lecithin phytocomplex group shows near-normal hepatic architecture with minimal lipid droplets and negligible inflammation. The graphs quantify steatosis, lobular inflammation, ballooning, and total NAS across treatment groups (excluding healthy controls), corresponding to the data presented in Table 2. Scale bar = 20 µm

The MASLD/MASH Activity Score (NAS) was calculated from standardized histological criteria, providing quantitative confirmation of hepatic damage and treatment efficacy across groups (Table 2). The MASLD control group exhibited the highest score, with a steatosis score of 3 (affecting > 66% of hepatocytes), a lobular inflammation score of 2–3 (multiple foci per 200 × field), and a ballooning score of 2 (prominent hepatocyte swelling), yielding a total score of 7–8, consistent with definite steatohepatitis. The fenofibrate-treated group showed moderate improvement, with steatosis reduced to 1–2 and fewer ballooned cells, resulting in a score of approximately 4–5, indicative of borderline MASH. The milk thistle-treated group displayed further reduction in lesion severity, with scores typically around 1 for steatosis and 0–1 for both inflammation and ballooning (total score: 2–3). Most notably, the group treated with the enhanced milk thistle formulation (with piperine and lecithin) achieved near-complete histological recovery, with minimal or absent steatosis, no ballooning, and little to no inflammatory infiltration (total score: 0–1), indicating resolution of steatohepatitis. As summarized in Table 2, histological evaluation demonstrated significant reductions in steatosis, lobular inflammation, and hepatocellular ballooning scores in all treated groups, with the most pronounced improvement observed in the silymarin–piperine–lecithin phytocomplex group (Fig. 5). These scoring outcomes align with both biochemical and microscopic findings, reinforcing the therapeutic potential of the enhanced phytocomplex in reversing MASLD pathology.

### PPAR-α immunohistochemistry reveals attenuation of oxidative DNA damage by enhanced milk thistle formulation in MASLD rats (Table 3, Fig. 6)

**Table 3:** The expression of PPAR-α protein in the liver tissue of the studied cohort

Group	H-score	p-value
Healthy Control	42 ± 3.1	
MASLD Control	246 ± 5.2	0.0001^**a**^
MASLD + Fenofibrate	136 ± 3.8	0.001^**a/b**^
MASLD + Milk Thistle	96 ± 3.5	0.004^**a/b**^
MASLD + Milk Thistle + Piperine + Lecithin	64 ± 4.2	0.02^**a/b**^

Values are presented as the mean of the H-score in each group. Superscripts denote significant differences: ^a^: Significant difference compared to the healthy control group (p < 0.05);^b^: Significant difference compared to the MASLD group (p < 0.05);a/b: Significant difference compared to both.

**Fig. 6:**
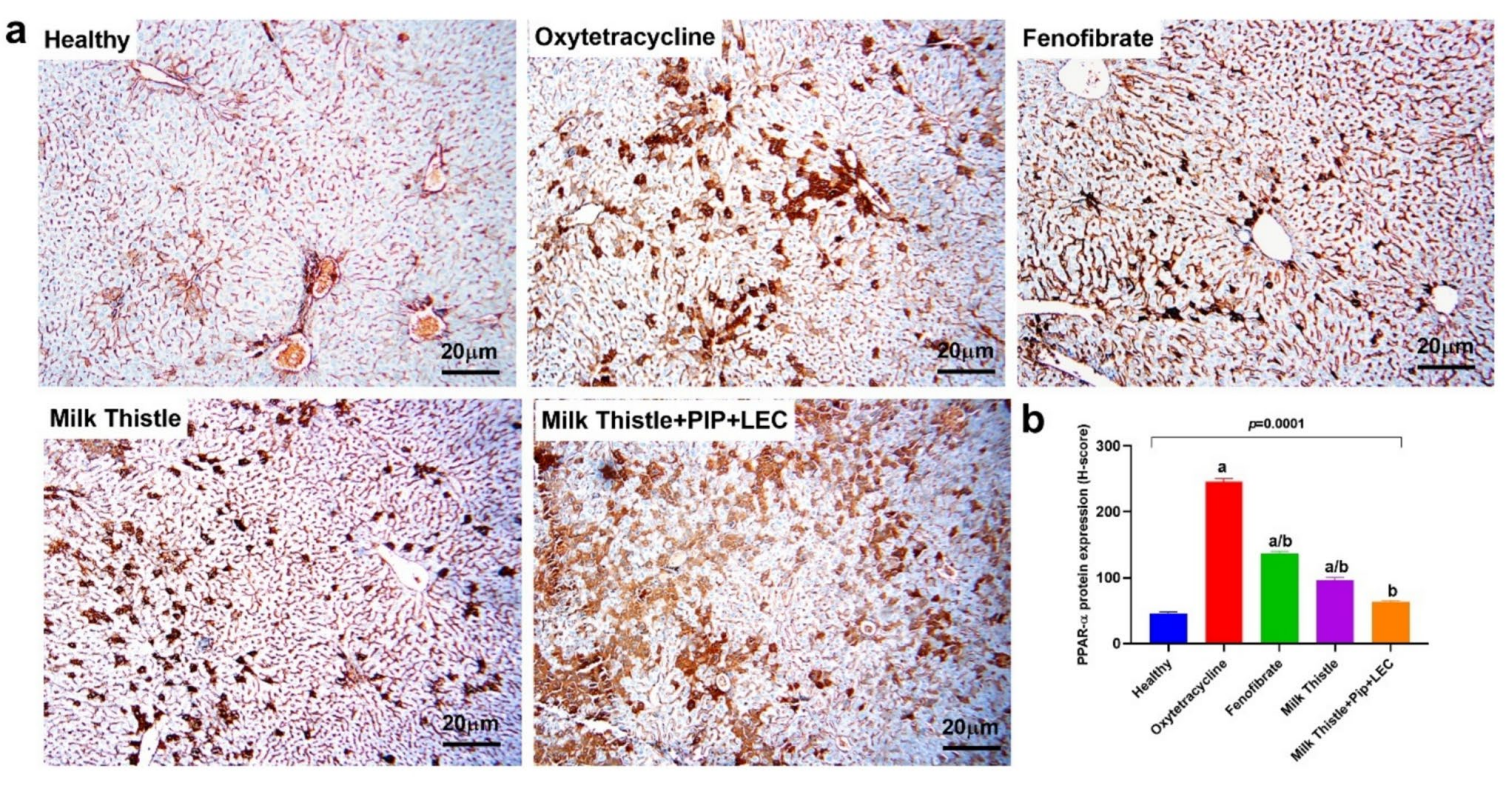
Immunohistochemical staining and quantification of PPAR-α expression in liver tissue from experimental groups. (**a**) Representative liver sections stained for PPAR-α (magnification × 10), including: Healthy control showing weak, baseline nuclear PPAR-α expression, Oxytetracycline-induced MASLD: exhibiting widespread strong nuclear staining, MASLD + Fenofibrate with moderate reduction in PPAR-Α-positive nuclei, MASLD + Milk Thistle showing further attenuation in staining intensity and distribution, and MASLD + Milk Thistle + Pipeline + Lecithin revealing minimal staining comparable to healthy tissue. (**b**) Quantitative analysis of PPAR-α expression across groups based on H-score. Data are expressed as mean ± SD (n = 5); statistical analysis was performed using one-way ANOVA followed by Tukey's post hoc test. Superscripts denote significant differences: ^a^: significantly different from healthy control (p < 0.05); = ^b^: significantly different from Oxytetracycline-induced MASLD (p < 0.05)

Immunohistochemical analysis of PPAR-α expression revealed distinct patterns across treatment groups (Table 3, Fig. 6a). The healthy control group showed minimal PPAR-Α immunoreactivity (H-score: 45.2 ± 4.1), consistent with physiological baseline expression. In contrast, the MASLD control group exhibited intense nuclear staining in a majority of hepatocytes, yielding a significantly elevated H-score of 235.6 ± 8.4 (p < 0.05 vs. healthy), reflecting increased oxidative stress and DNA repair activity associated with hepatocellular damage.

Treatment with fenofibrate led to a moderate decrease in PPAR-α staining (H-score: 138.3 ± 6.7), while milk thistle extract further attenuated expression (H-score: 96.8 ± 5.3), indicating reduced hepatic stress. Notably, the combination treatment of milk thistle with piperine and lecithin produced a marked downregulation of PPAR-α expression (H-score: 58.7 ± 4.8), approaching levels observed in the healthy control group (Table 3, Fig. 6b). These findings suggest that the enhanced formulation provides superior protection against MASLD-associated oxidative DNA damage, likely due to improved bioavailability and synergistic antioxidant effects.

## Discussion

Metabolic dysfunction-associated steatotic liver disease (MASLD) is a burgeoning global health issue, currently affecting approximately one-quarter of the world's population (Albeshry et al. 2023; Younossi et al. 2023). Despite increasing recognition of its clinical burden, effective pharmacological treatments remain limited (Younossi and Henry 2024), and there is an urgent need for safer, natural, and efficacious therapeutic alternatives (Ofosu et al. 2018). While silymarin, a flavonolignan-rich extract from *Silybum marianum,* has been widely studied for its hepatoprotective properties, its clinical utility has been hindered by poor bioavailability (Freitag et al. 2015; Jiang et al. 2022a, b). Additionally, bioenhancers like piperine and phospholipid-based carriers such as lecithin have been explored in other contexts to improve the pharmacokinetics of phytochemicals (Arora et al. 2023; Jacob et al. 2025), but the combined impact of these three agents on MASLD has not been thoroughly investigated. This study sought to address that gap by evaluating the efficacy of a novel phytocomplex formulation comprising silymarin, piperine, and lecithin, compared to fenofibrate, in an established rat model of MASLD induced by oxytetracycline.

The study design included well-defined histological, biochemical, and immunohistochemical assessments, enabling a multi-tiered evaluation of hepatocellular injury, lipid metabolism, and oxidative DNA damage. The results demonstrate that the enhanced silymarin phytocomplex markedly improves hepatic function, restores lipid profile, reduces hepatic fat accumulation, normalizes histopathological architecture, and significantly downregulates PPAR-α, a marker of oxidative DNA stress, surpassing the effects of both crude silymarin extract and fenofibrate.

The dramatic reduction in liver enzymes observed with the enhanced phytocomplex aligns with previous clinical studies demonstrating silymarin's hepatoprotective effects. A systematic review by Zhang et al. (2023) found that silymarin significantly reduced ALT (MD = −17.12) and AST (MD = −12.56) levels in MASLD patients (Malik et al. 2024). Similarly, clinical trials have shown that silymarin-containing formulations effectively decreased liver enzymes and improved lipid parameters in MASH patients (Curcio et al. 2020; Jaffar et al. 2024a, b). Fenofibrate, a known PPAR-α agonist, partially reduced these enzymes, confirming its lipid-modifying and anti-inflammatory roles in hepatic tissue (Fan et al. 2024). However, both the silymarin-treated and particularly the phytocomplex-treated groups exhibited more pronounced normalization of liver enzymes, indicating a superior hepatocellular protective effect. The findings corroborate earlier reports on silymarin's membrane-stabilizing and antioxidant roles, yet notably extend them by demonstrating how lecithin and piperine significantly potentiate these effects (Aghemo et al. 2022).

Lipid profile parameters further supported this therapeutic benefit. MASLD rats showed classic dyslipidemia, elevated total cholesterol, triglycerides, LDL, and VLDL, with reduced HDL levels. Fenofibrate partially corrected these abnormalities, consistent with its approved clinical use in dyslipidemia. However, the phytocomplex achieved near-complete normalization, including HDL restoration, suggesting a multifaceted mechanism beyond PPAR-α activation likely encompassing silymarin's antioxidant modulation of lipogenesis and piperine's enhancement of hepatic enzyme activity.

The superior lipid profile restoration achieved by the phytocomplex, including normalization of HDL (47.6 ± 1.94 mg/dL) and LDL (38.2 ± 4.88 mg/dL), corresponds with findings from nano-formulation studies where enhanced bioavailability led to improved therapeutic outcomes (Liang et al. 2018). The atherogenic index normalization (2.29 ± 0.16) suggests potential cardiovascular protective benefits, which is particularly relevant given the close association between MASLD and metabolic syndrome (Ismaiel et al. 2022).

Histologically, the H&E-stained sections and NAS scoring provided further compelling evidence. The MASLD model group displayed hallmark features of MASH macrovesicular steatosis, hepatocellular ballooning, lobular inflammation, and portal fibrosis closely mimicking human pathology. Fenofibrate-treated livers showed partial resolution, particularly in lipid vacuolization, though signs of ongoing inflammation and fibrosis persisted. Treatment with crude milk thistle improved hepatocyte morphology and reduced both fat accumulation and inflammation, yet mild fibrosis and ballooning remained evident. Notably, only the group treated with the phytocomplex formulation showed near-complete histological normalization: fat vacuoles were nearly absent, lobular inflammation was minimal, and hepatocytes displayed normal architecture and nuclear morphology. These findings highlight the enhanced efficacy of the phytocomplex and reinforce previous work suggesting the importance of improving silymarin bioavailability to unlock its full therapeutic potential (Di Costanzo and Angelico 2019).

Immunohistochemical analysis of PPAR-α expression in this study revealed its marked downregulation in the MASLD group, aligning with reports that hepatic steatosis suppresses PPAR-α activity, impairing β-oxidation and lipid clearance (Pan et al. 2022; Todisco et al. 2022). Treatment with fenofibrate, a known PPAR-α agonist, restored its nuclear expression, consistent with its mechanism of action (Qiu et al. 2023; Soukop et al. 2025). Similarly, both silymarin and the phytocomplex significantly upregulated PPAR-α, supporting previous findings that silymarin enhances lipid metabolism via PPAR-α modulation (Guo et al. 2024). The phytocomplex group showed expression levels comparable to fenofibrate, suggesting that bioenhanced silymarin may exert comparable PPAR-α activation, offering a natural alternative for correcting MASLD-associated metabolic dysfunction.

This study presents several novel aspects that distinguish it from previous research. The combination of silymarin with both lecithin (phytosome technology) and piperine (bioenhancement) represents an innovative approach to addressing bioavailability limitations that have historically restricted natural product therapeutics. While individual studies have explored silymarin-lecithin complexes (Gillessen and Schmidt 2020) or piperine bioenhancement separately (Ralli et al. 2023), this research demonstrates the synergistic potential of combining both strategies.

This study has several limitations that should be considered. The 90-day duration, while suitable for assessing short-term efficacy, may not fully reflect the chronic progression of MASLD and MASH, which typically evolve over months to years. In addition, the relatively small sample size (n = 5 per group) may limit statistical power and the detection of subtle intergroup differences, and the use of the oxytetracycline model induces steatosis efficiently but lacks full translational relevance to human MASLD pathology. Furthermore, the absence of pharmacokinetic and bioavailability assessments for silymarin, piperine, and lecithin restricts the mechanistic interpretation of their synergistic effects. Future studies employing larger cohorts, extended treatment durations, and pharmacokinetic profiling are warranted to confirm and expand upon these findings.

Despite these limitations, the findings offer promising avenues for future exploration. Clinical translation could involve pilot trials assessing the bioavailability, safety, and efficacy of the phytocomplex in human subjects with early-stage MASLD. Furthermore, formulation into capsules or functional foods may provide patient-friendly therapeutic options. Longitudinal studies assessing fibrosis regression, insulin sensitivity, and gut–liver axis modulation will be critical in validating these preliminary results.

## Conclusion

In conclusion, this study demonstrates that a novel formulation of silymarin enhanced with piperine and lecithin significantly attenuates liver injury, lipid dysregulation, and oxidative stress in MASLD. This phytocomplex not only outperforms crude silymarin but also exhibits superior efficacy to fenofibrate, a current standard treatment. These findings highlight the therapeutic promise of optimized natural compounds in managing complex metabolic liver disorders and advocate for further preclinical and clinical development.

## Data Availability

The data analyzed during this study can be obtained from the corresponding author upon a reasonable request.

## Abbreviations

MASLD: Metabolic Dysfunction-Associated Steatotic Liver Disease
MASH: Metabolic Dysfunction-Associated Steatohepatitis
ALP: Alkaline Phosphatase
ALT: Alanine Transaminase
AST: Aspartate Transaminase
DAB: Diaminobenzidine
H&E: Hematoxylin And Eosin
HCC: Hepatocellular Carcinoma
HDL: High Density Lipoprotein
HRP: Horseradish Peroxidase
H-Score: Histo Score H
IHC: Immunohistochemical
LDL: Low Density Lipoprotein
NAS: MASLD/MASH Activity Score
PBS: Phosphate Buffered Saline
PPAR-Α: Peroxisome Proliferator Activated Receptor Alpha PPAR
PV: Portal Vein
SD: Standard Deviation
V/W: Solvent To Solid Ratio V
